# Supramolecular Organocatalysis in Water Mediated by Macrocyclic Compounds

**DOI:** 10.3389/fchem.2018.00084

**Published:** 2018-04-03

**Authors:** Margherita De Rosa, Pellegrino La Manna, Carmen Talotta, Annunziata Soriente, Carmine Gaeta, Placido Neri

**Affiliations:** Dipartimento di Chimica e Biologia “ Zambelli”, Università degli Studi di Salerno, Salerno, Italy

**Keywords:** supramolecular organocatalysis, water, hydrophobic effect, molecular recognition, calixarenes, cyclodextrins

## Abstract

In the last decades many efforts have been devoted to design supramolecular organocatalysts able to work in water as the reaction medium. The use of water as solvent provides promising benefits with respect to environmental impact. In this context, macrocyclic compounds played a role of primary importance thanks to their ease of synthesis and their molecular recognition abilities toward the reactants. The aim of this review is to give an overview of the recent advances in the field of supramolecular organocatalysis in water, focusing the attention on calixarene and cyclodextrins derivatives. Calixarenes and cyclodextrins, thanks to their hydrophobic cavities, are able to host selectively the substrates isolating they from the reaction environment. In addition, the synthetic versatilities of these macrocycles permits to introduce useful functional groups in close proximity of the hydrophobic binding sites. Regarding the cyclodextrins (CDs), we have here reviewed the their most recent uses as organocatalysts for the synthesis of heterocyclic compounds, in multi-component reactions and in carbon-carbon bond forming reactions. Examples have been reported in which CD catalysts are able to drive the regiochemistry of common organic reactions. In addition, cyclodextrins bearing catalytically active chiral groups, have shown excellent enantioselectivity in the catalysis of organic reactions. Recently reported results have shown that calixarene derivatives are able to accelerate organic reaction under “on-water” conditions with a significant selectivity toward the reactants. Under “on-water conditions” the hydrophobic effect, induced by insoluble calixarene derivatives, forces the reactants and the catalyst to aggregate and thus accelerating the reaction between them thanks to an amplification of weak secondary interactions. Regarding the use of water-soluble calixarene organocatalysts, we have here reviewed their role in the acceleration of common organic reactions.

## Introduction

In the last decades, a growing interest has been devoted to the development of new synthetic methodologies with the aim to emulate the high performance of biological processes (Dong et al., 2012; Raynal et al., 2014; Borsato and Scarso, 2016; Deraedt and Astruc, 2016; Kuah et al., 2016). In natural biosynthetic processes, the enzymatic machines work in water where they reach amazing levels of efficiency, selectivity, and specificity (Copeland, 2002; Bugg, 2012). Taking inspiration from the biological systems, several scientists have devoted many efforts to the design of artificial systems with the aim of mimicking the levels of performance of natural enzymes. At this regards, a particular emphasis has been placed to the study of organic reactions in water as the medium (Kobayashi and Li, 2012). Water, as a solvent, possesses many desirable characteristics for a reaction medium, because it is not only environmentally benign, but with its high heat capacity, high polarity, large cohesive energy, and hydrogen bonding abilities may have a significant impact on the reaction course influencing reaction rate and selectivity. In a pioneering work, Breslow highlighted the attractiveness of water as reaction medium and as reaction promoter of a Diels-Alder cycloaddition (Rideout and Breslow, 1980; Breslow, 1991; Simon and Li, 2012). Successively, Sharpless introduced for the first time the expression “on-water conditions” to indicate the rate acceleration observed in organic reactions when insoluble reactants are vigorously stirred in a water suspension (Narayan et al., 2005; Pirrung, 2006). As shown by Schreiner (Kleiner and Schreiner, 2006) and Rueping (Rueping and Theissmann, 2010), the polar nature and the hydrogen bonding abilities of water do not limit its use as a solvent in organocatalyzed reactions where the substrates are activated by covalent or secondary weak-interactions (Sakthivel et al., 2001; Pirrung and Sarma, 2004; Pirrung, 2006; Raj and Singh, 2009; Butler and Coyne, 2010; Mase and Barbas, 2010; Giacalone and Gruttadauria, 2013; De Rosa et al., 2016, 2017b; Jimeno, 2016). On the other hand, in fact, the natural enzymes stay and work in aqueous environment, where the enzyme-substrate complexes survive to the presence of water. The knowledge of the mechanisms underlying enzymatic catalysis has been an important source of inspiration for the design of supramolecular biomimetic organocatalysts (Steed and Atwood, 2009; Kirby and Hollfelder, 2010; Marchetti and Levine, 2011; Raynal et al., 2014). Thus, in the last years different types of supramolecular organocatalysts have been designed which have shown excellent catalytic efficiency and amazing regio-and stereoselectivity (Zhang and Tiefenbacher, 2015; Zhang et al., 2017; La Manna et al., 2018). The supramolecular organocatalysts so far known are constituted by macrocyclic scaffolds in which an internal cavity binds the substrate through secondary interactions with functional groups present in close proximity of the cavity. Inspired by the natural systems, in the macrocyclic-based supramolecular organocatalysts the internal cavity is able to accommodate the reactants isolating them from the bulk medium and provides a confined reaction environment. In this way, an enhancement of the local concentration of the reactants is obtained, thus promoting the reaction by proximity effects. In addition, the substrate selectivity is dictated by the size and shape of the internal cavity. More recently, many efforts have also been devoted to the design of self-assembled nanocapsules able to host different guests and to promote reactions inside their cavities. (Dong et al., 2011, 2012; Longstreet and McQuade, 2013; La Sorella et al., 2015; Deraedt and Astruc, 2016; Kuah et al., 2016).

**Figure 1:**
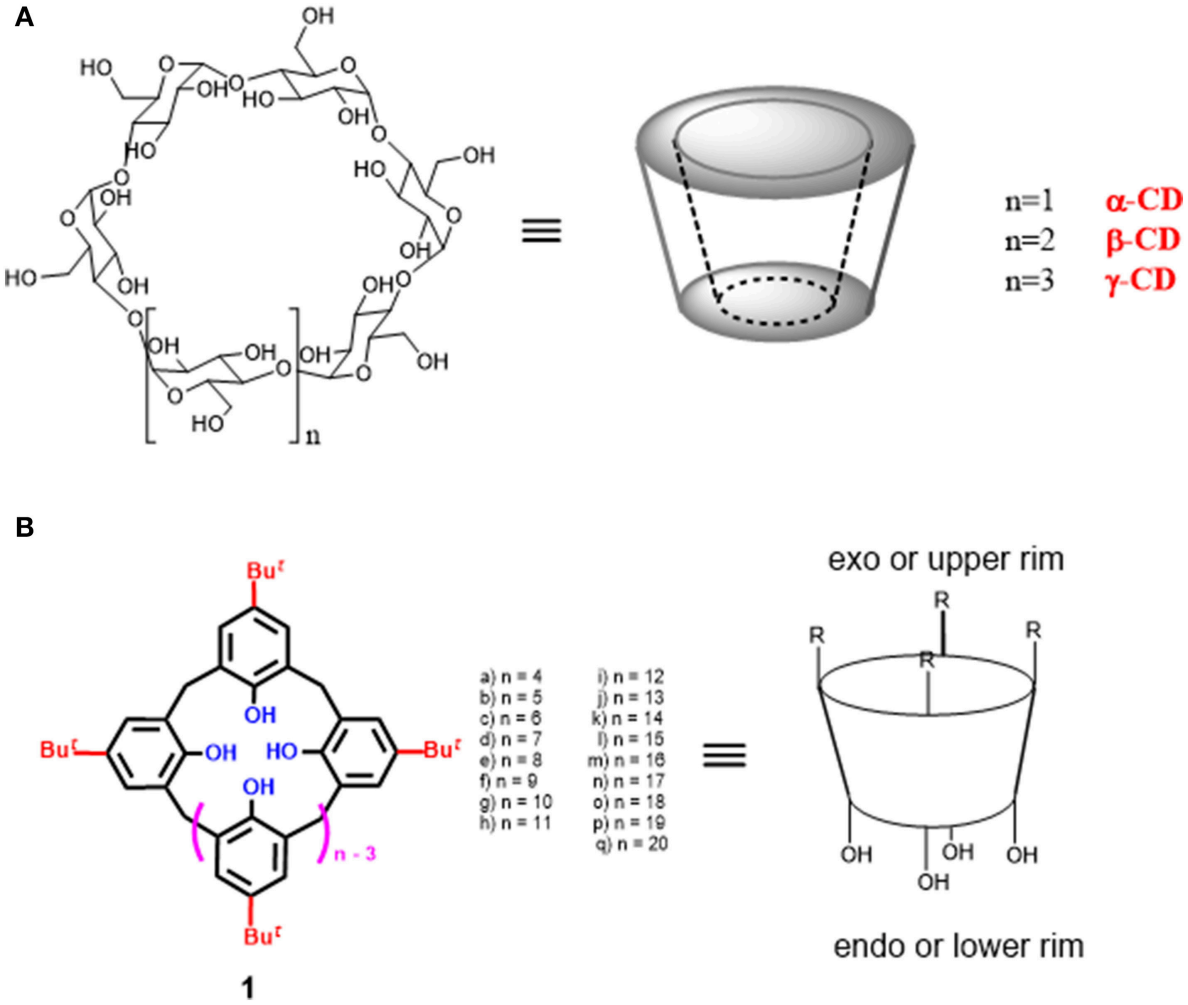
Chemical structure of macrocyclic cavity-containing scaffolds: **(A)** cyclodextrins (CD); **(B)** calix[n]arenes.

In this review, we highlight some recent examples of macrocyclic-based supramolecular organocatalysts (Figure 1) able to catalyze organic reactions using water as a reaction medium. In details, we will focus our attention on examples concerning the use of calixarene and cyclodextrin macrocycles as scaffolds for supramolecular catalysts. Calixarenes and cyclodextrins are among the most popular macrocycles in supramolecular chemistry where they have shown amazing abilities in different fields. Calixarene and cyclodextrin macrocycles possess many desirable features for the design of supramolecular catalysts: (a) a hydrophobic internal cavity able to host the substrates in a selective way; (b) an excellent synthetic versatility which permits the introduction of functional groups in close proximity of their cavities. Thanks to these characteristics, calixarene, and cyclodextrin derivatives have found many applications in the field of supramolecular organocatalysis and we will highlight the most recent ones in this review.

In Tables 1, 2 the most recent catalytic performances of cyclodextrins and calixarenes evaluated in this work are summarized.

**Table 1:** Summary of the catalytic performance of cyclodextrin macrocycles evaluated in this review.

**Organocatalyst**	**Application**	**References**
Native β-CD	Synthesis of tryptanthrin analogs	Kumar et al., 2011
	Synthesis of 2,3-dihydroquinazolin-4(1H)-one derivatives via three-component reaction	Ramesh et al., 2012
	C-H bond functionalization of 2-alkyl-azaarenes with diones	Kumar and Shukla, 2014
	Deprotection of hydroxyl group accompanied by cyclization or ring opening of chalcone epoxides	Kumar et al., 2015
	Synthesis of polysubstituted pyrroles via one-pot four component procedure	Konkala et al., 2016
	1,3-dipolar cycloaddition	Floresta et al., 2017
Native β-CD /IBX	Deprotection with oxidative cleavage of chalcone epoxides and oxidative dehydrogenation of alcohols	Kumar and Ahmed, 2016
**MODIFIED CYCLODEXTRINS**		
Proline-cyclodextrin coniugates	Aldol reaction	Doyagüez and Fernández-Mayoralas, 2012
Proline-cyclodextrin coniugates	Aldol reaction	Liu and Zhang, 2015
β-cylodextrins peramino	One-pot synthesis of enantiomerically enriched quinolone derivatives	Kanagaraj and Pitchumani, 2013
Sulfonated-β-cyclodextrin	Electrophilic substitution reaction of indoles with isatins	Tayade et al., 2015
Partially methylated β-CDs (RAME-β-CD)	Paal-Knorr condensation between 1,4-diketones and primary amines	Menuel et al., 2014 Akelis et al., 2016

**Table 2:** Summary of the catalytic performance of calixarene macrocycles evaluated in this review.

**Organocatalyst**	**Application**	**References**
Calix[4]arene with a chiral proline moiety at the upper or lower rim	Aldol reaction	Li et al., 2009
		Li et al., 2010
		Eymur et al., 2014
		Uyanik et al., 2014
		Aktas et al., 2016
		Sahin et al., 2016
		Li et al., 2017
Calix[4]arene with imidazole moieties at the lower rim	Aromatic nucleophilic substitution reaction Benzyl nucleophilic substitution	Yang et al., 2012
Calix[n]-arenes with quaternary ammonium salts at the upper rim	One-pot Mannich reaction	Sayin and Yilmaz, 2014
p-Sulfonic acid calixarenes	Synthesis of 2,3-dihydroquinazolyn-4(1H)-ones	Rahaman et al., 2015
Calix[6]arene sulfonic acid derivatives	Michael addition between indoles and α,β-unsaturated ketones	Xie et al., 2013
Thioureido calix[n]arenes	Vinylogous Mukaiyama Aldol reaction	De Rosa et al., 2016
Tetraminocalix[4]arene	Vinylogous Mukaiyama Aldol reaction	De Rosa et al., 2017b

## Reaction organocatalyzed by cavity-containing macrocyclic scaffolds

### Cyclodextrins

Cyclodextrin macrocycles (CD) are a family of naturally occurring molecules formed by α-1,4-linked D-glucose units associated head-to-tail to form a truncated conical structure. They have a hydrophobic inner core, made by glycosidic oxygen atoms and hydrocarbon CH-groups, which is surrounded by an outer hydrophilic surface formed by polar hydroxyl groups. The presence of hydroxyl groups ensures not only their water solubility but also the possibility of a direct interactions with substrates or the introduction of other functional groups. The number of the glucopyranoside units in the macrocycle modulates the cavity size being the most common cyclodextrins composed of 6 (α-CD), 7 (β-CD), and 8 (γ-CD) units (Figure 1).

The first evidences for the potential use of CDs in catalysis as enzyme models can be traced back to Bender's studies in the hydrolysis of phenyl acetate in the presence of CD where a remarkable substrate specificity was found (Van Etten et al., 1967a,b). Since then, a large number of works have been reported regarding the role of native and modified CDs in different types of reactions (Macaev and Boldescu, 2015; Srivastava et al., 2016; Bai et al., 2017). Their catalysis may be classified generally in two types: (a) covalent when an CD -reactant intermediate is formed through a covalent bond which then evolves in products, (b) non-covalent when the interactions between CD and reactants are non-covalent and CD hydrophobic cavity offers a microenvironment where the reaction takes place (Breslow and Dong, 1998; Takahashi, 1998; Komiyama and Monflier, 2006). Interestingly, complex of the CDs with transition metal have been used as water-soluble organometallic catalysts in several reactions, in addition CDs based material have been used as unconventional reaction media such as supramolecular hydrogels or low melting mixtures (LMMs) (Hapiot et al., 2014, 2017). Furthermore, when metals are not involved in the CD catalytic activity, the catalysis falls in the field of the supramolecular organocatalysis (Bogliotti and Dalko, 2007; Marinescu and Bols, 2010), and interesting works have been reported in recent years.

#### Native cyclodextrins promoted reactions

**Figure 2:**
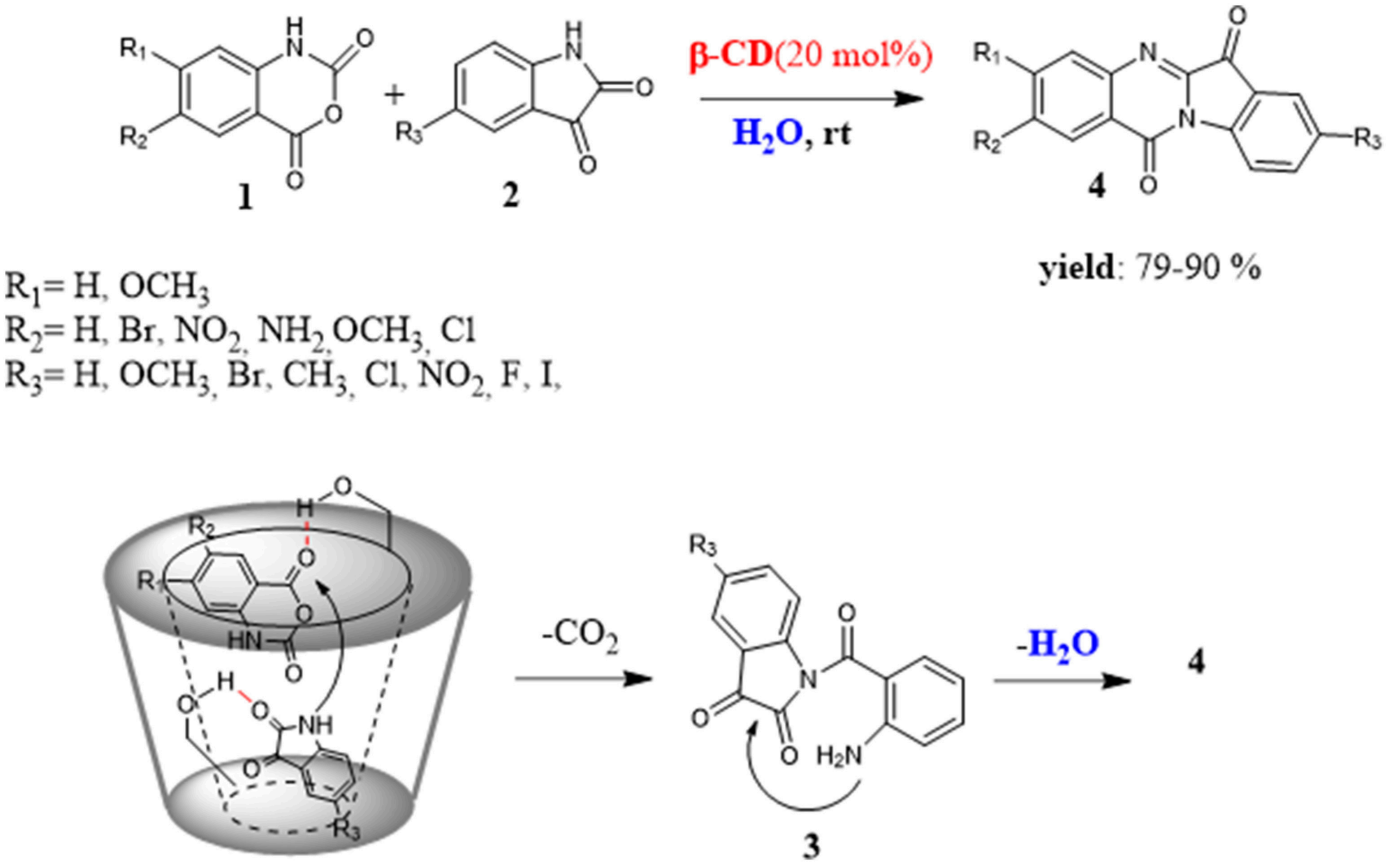
CD mediated synthesis of tryptanthrin analogs in water.

In 2011, Kumar et al. reported an efficient and eco-friendly procedure for the synthesis of tryptanthrin analogs **4** mediated by non-modified β-CD (Kumar et al., 2011). Tryptanthrin analogs are an interesting class of heterocycle compounds characterized by a broad spectrum of biologic activities against different pathogens such as antibacterial, antifungal, antitubercular activities and, furthermore a potential anticancer agent against some human cancer cell-lines (Dzierzbicka et al., 2003; Martinez-Viturro and Dominguez, 2007; Kshirsagar, 2015). The reaction did not proceed without the assistance of CD and, between the most common cyclodextrins, only β-CD proved to be the most efficient catalyst. These results pointed out the dual role played by the CD not only as catalyst but also as nanoreactor; the β-CD had the right cavity size to accommodate both reactants and thus induce the reactive events. The mechanistic hypothesis for the reaction proposed the imprisonment of the reactants in the CD cavity, the activation of carbonyl group at C-4 position of isatoic anhydride **1** through hydrogen bonding interaction with the hydroxyl groups of the CD, attack of the isatin **2** leading to cleavage of anhydride ring and formation of an intermediate **3** which, in turn, transforms into the final product **4**. (Figure 2) ^1^H-NMR studies confirmed the formation of an inclusion complex between isatoic anhydride **1** and β-CD. The substrate scope proved general with different substituted isatins as well as substituted isatoic anhydrides affording the product in good to high yields not in a long time (5–9 h).

**Figure 3:**
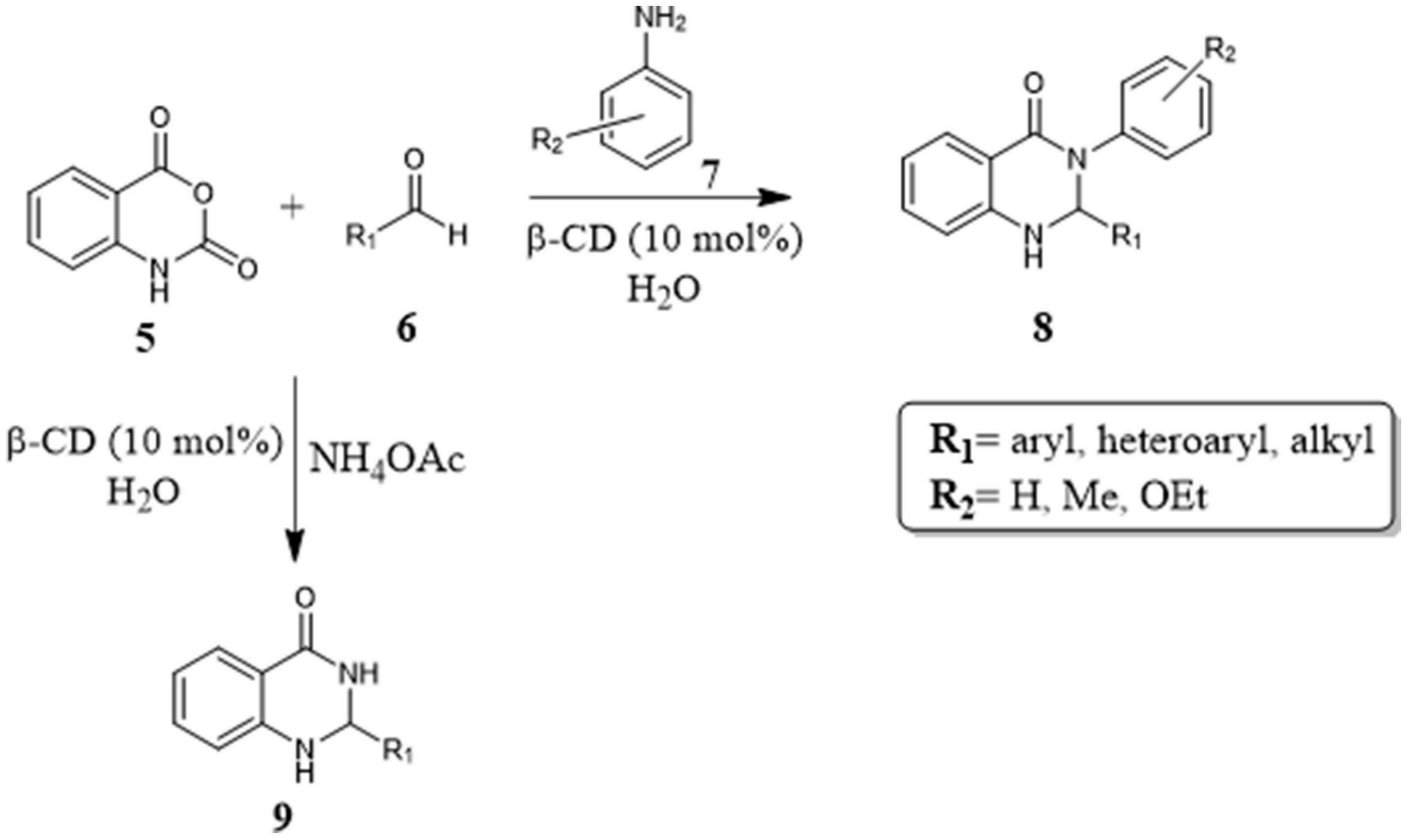
CD promoted one-pot synthesis of 2,3-dihydroquinazolin-4(1H)-one derivative.

In 2012, Ramesh and coworkers reported an interesting example of a one-pot protocol mediated by CDs in water. (Figure 3) For the first time a cyclodextrin promoted via three-component reaction the synthesis of 2,3-dihydroquinazolin-4(1H)-one derivatives **8** and **9**, molecules exhibiting several biological and pharmacological activities. The reaction between aniline **7**, aldehyde **6** and isatoic anhydride **5** afforded the products in good yields and in short reaction times with only 10%mol β-CD. The recovery and reuse of the CD with little loss of efficiency, simple work-up and the use of water as reaction medium made it a powerful alternative method. (Ramesh et al., 2012).

**Figure 4:**
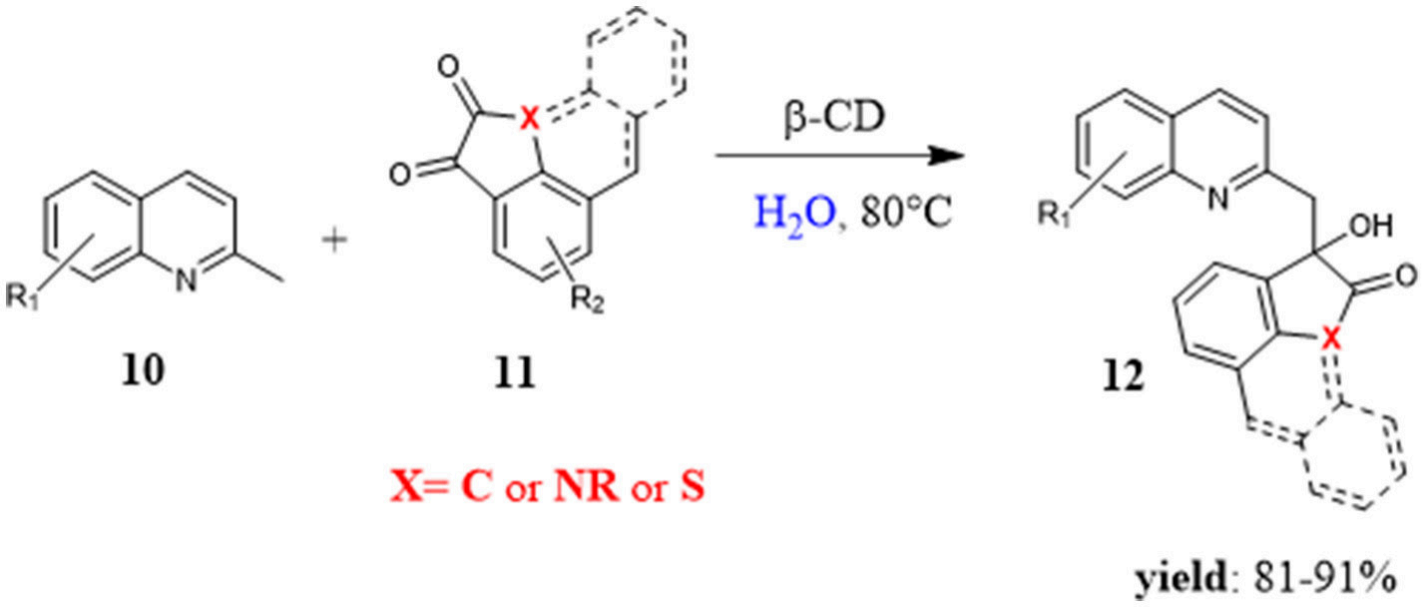
β-CD mediated C(sp^3^)-H functionalization of 2-alkyl-azaarenes with diones in water.

In 2014, Kumar and Shukla used cyclodextrins as catalysts to perform a C(sp^3^)-H functionalization of 2-alkyl-azaarenes **10** with a series of homocyclic and heterocyclic diones **11** in water. (Figure 4) The reaction was interesting because it was a simple, green, and valid route to form carbon-carbon and carbon-heteroatom bonds, generally formed in the presence of transition metals. Different CDs were evaluated, β-CD turned out to be the best catalyst affording the product **12** in high yield in only 4 h at 80°C. The catalytic role of CD was pointed out by the absence of reactivity without CD. Studies by ^1^H-NMR spectra underlined up field shift of β-CD protons when mixed with the reactants thus confirming the formation of inclusion complexes. Under the optimal reaction conditions, the reaction was general for various isatins and 2-methylquinoline, different heterocyclic and homocyclic diones, regardless of the substitution pattern. Interestingly, the catalyst was recyclable up to 5 times without significant losses of efficiency (Kumar and Shukla, 2014).

**Figure 5:**
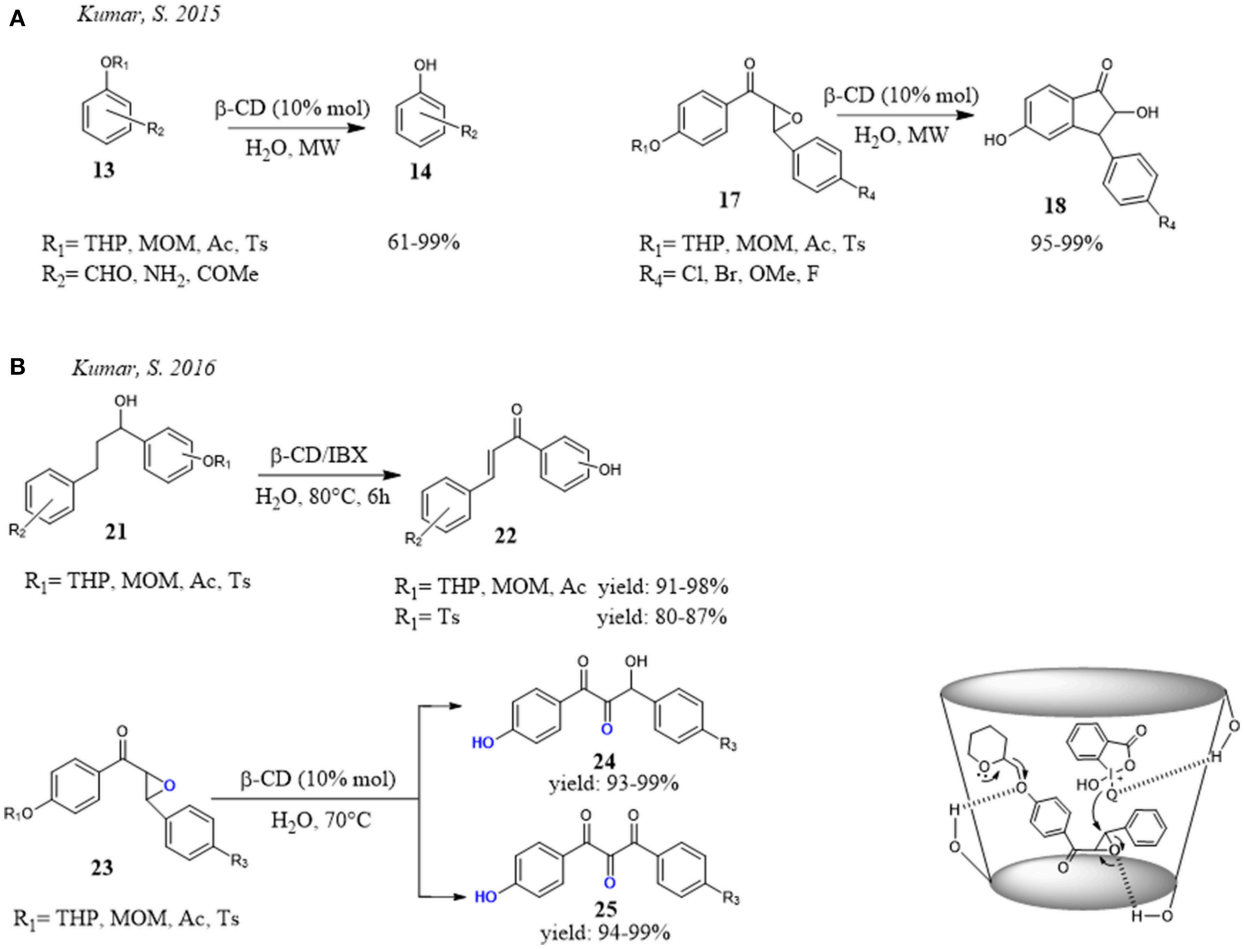
β-CD mediated deprotection of hydroxyl groups in water: **(A)** deprotection reaction and one-pot deprotection with subsequent cyclization of chalcone epoxides; **(B)** one-pot deprotection and concurrent oxidation reaction.

Interesting and promising application of CD was proposed by Kumar et al. for the deprotection of hydroxyl groups. (Figure 5) They reported that THP/MOM/Ac/Ts ethers **13** could be deprotected under mild and eco-friendly conditions in the presence of β-CD in water. Particularly, the combined use of water and microwave irradiations greatly reduced the reaction times to minutes without loss of efficiency (Figure 5A). It is noteworthy that, under the reaction conditions, they observed not only deprotection of hydroxyl group but also a concomitant regioselective cyclization of chalcone epoxides **17** to 2-hydroxyindanones **18** and 2′-aminochalcones to aza-flavanones, respectively. Different evidence was recorded for the chalcones, the procedure stopped at the deprotection without evidence of cyclization. With 2′-aminochalcones the reaction efficiency depended on the nature of substituents. If the substituent on Ar^2^ ring of aminochalcones was electron-donating group, the cyclization was favored giving the product in higher yield, while an electron-withdrawing group slowed the cyclization and so decreased the reaction efficiency. In the reaction mechanism proposed by the authors, the CD played a dual role: (a) activation of the THP ether and epoxide by H-bond interactions and, at the same time (b) formation of inclusion complex controlled the regioselective ring opening of epoxide at β-carbon. (Kumar et al., 2015). Later, the authors extended this procedure (Kumar and Ahmed, 2016). The reaction of deprotection was carried out using β-CD and o-iodoxybenzoic acid (IBX) in water. Under these conditions a one-pot deprotection with oxidative cleavage of chalcone epoxides **23** and oxidative dehydrogenation of alcohols **21** was observed. The reaction afforded β-hydroxy-1, 2-diketones **24**, α,β-unsaturated ketones **22** and 1,2,3-triketones **25** in moderate to high yields requiring low reaction time. Once again, the dual role played by β-CD was evident. In the THP ether deprotection and concurrent oxidative cleavage of chalcone epoxide, β-CD not only activated the ether and epoxide through hydrogen bonding interactions, but also controlled the regioselective opening of epoxide forming inclusion complex (Figure 5).

**Figure 6:**
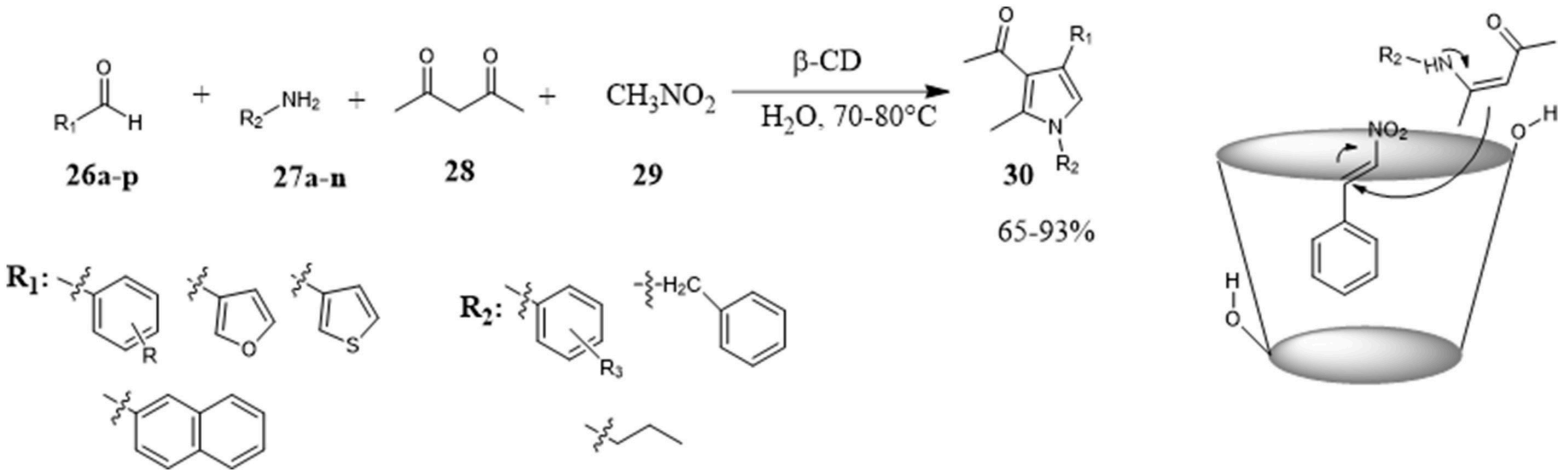
One-pot four-component procedure for the synthesis of substituted pyrroles in water promoted by β-CD.

One-pot four-component procedure promoted by CD for the preparation of polysubstituted pyrroles **30** was reported for the first time by Konkala et al. (2016) The synthesis of substituted pyrroles is complicated mainly by the occurrence of undesired polymerization reactions or lack selectivity, this protocol provided efficient and selective. The poor results obtained by using sodium dodecyl sulfate (SDS) or PEG-400, pointed out that CD did not act as a transfer phase catalyst and so the high reaction performance in its presence could be attributed to the formation of inclusion complexes within its hydrophobic cavity thereby facilitating the sequence of the reaction. NMR studies confirmed the incorporation of the aldehyde **26** and the formation of β-CD-aldehyde inclusion complex. Once activated, the aldehyde reacted with nitromethane **29** to form nitrostyrene which, in turn, reacted with *in situ* generated β-ketoenamine and, a subsequent oxidative aromatization led to the final product. (Figure 6) The scope of the reaction is broad with respect to the nature of the aldehyde and amine affording many substituted pyrroles in good to high yields.

**Figure 7:**
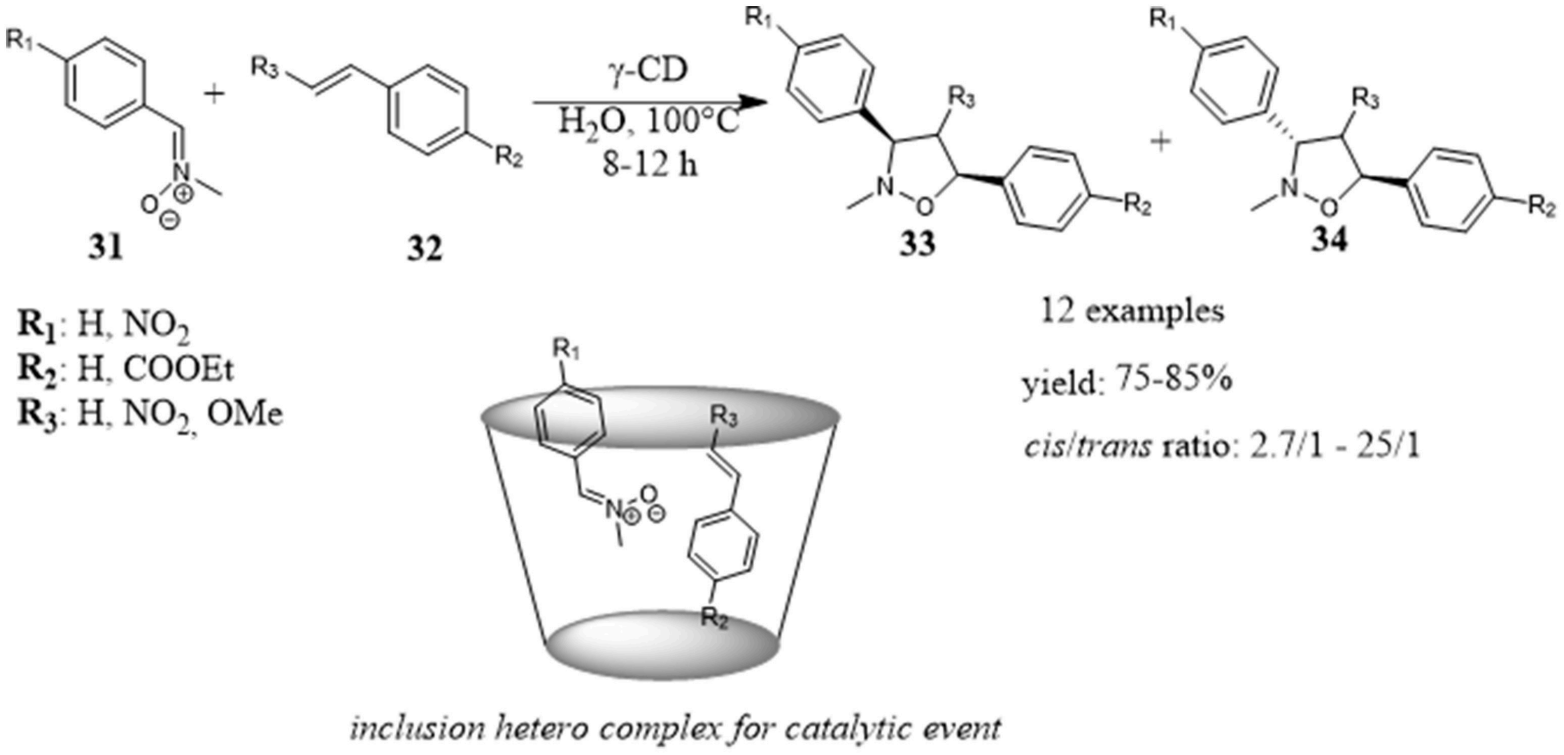
γ-Cyclodextrin catalyzes 1, 3-dipolar cycloaddition between different nitrones and styrenes to afford substituted isoxazolidines.

Recently, Floresta et al. employed γ-cyclodextrin as a catalyst in the 1, 3-dipolar cycloaddition between different nitrones **31** and styrenes or cynnamates **32** to give substituted isoxazolidines **33** and **34** in high yields and good to high diastereomeric excesses. The role of CD was ascribed to the formation of host-guest complexes between CD and the reactants mediated by noncovalent interactions (Figure 7). When the reactants were included together into the hydrophobic CD cavity, the catalytic event took place. In fact, the reaction did not proceed with α- and β-CD, their cavity size were too small to simultaneously accommodate both reactants. Evidences from HR FT-ICR MALDI-MS and NMR studies supported these conclusions. The recycle of catalyst without loss of activity, the use of water as reaction medium and high reaction efficiency made the protocol competitive with those carried out in organic solvent. Furthermore, *in silico* studies were used to rationalize the reaction stereoselectivity outcome and to improve it by the choice of proper substrates able to interact with the cyclodextrin through hydrogen bond interactions. In fact, the calculations supported an inclusion mode with the insertion of phenyl ring of styrene **32** within the cavity pointing to the down rim and with the phenyl of nitrone **31** protruding out the cavity at the height of secondary OH groups in the upper rim (Floresta et al., 2017).

#### Modified cyclodextrins promoted reactions

Many modified cyclodextrins have been prepared through selective conversion of the hydroxyl groups to other functionalities with the aim to modify and improve their catalytic abilities. The introduction of one or more reactive binding sites in precise positions can offer multiple molecular recognition sites and allow a more effective and selective catalysis (Hattori and Ikeda, 2006).

In the field of supramolecular organocatalysis, the rationale of this approach is based on the possibility of combining the catalytic properties of an organocatalyst placed in an appropriate position on the CD scaffold and suitable for the target reaction and the ability of cyclodextrins to form inclusion complexes.

**Figure 8:**
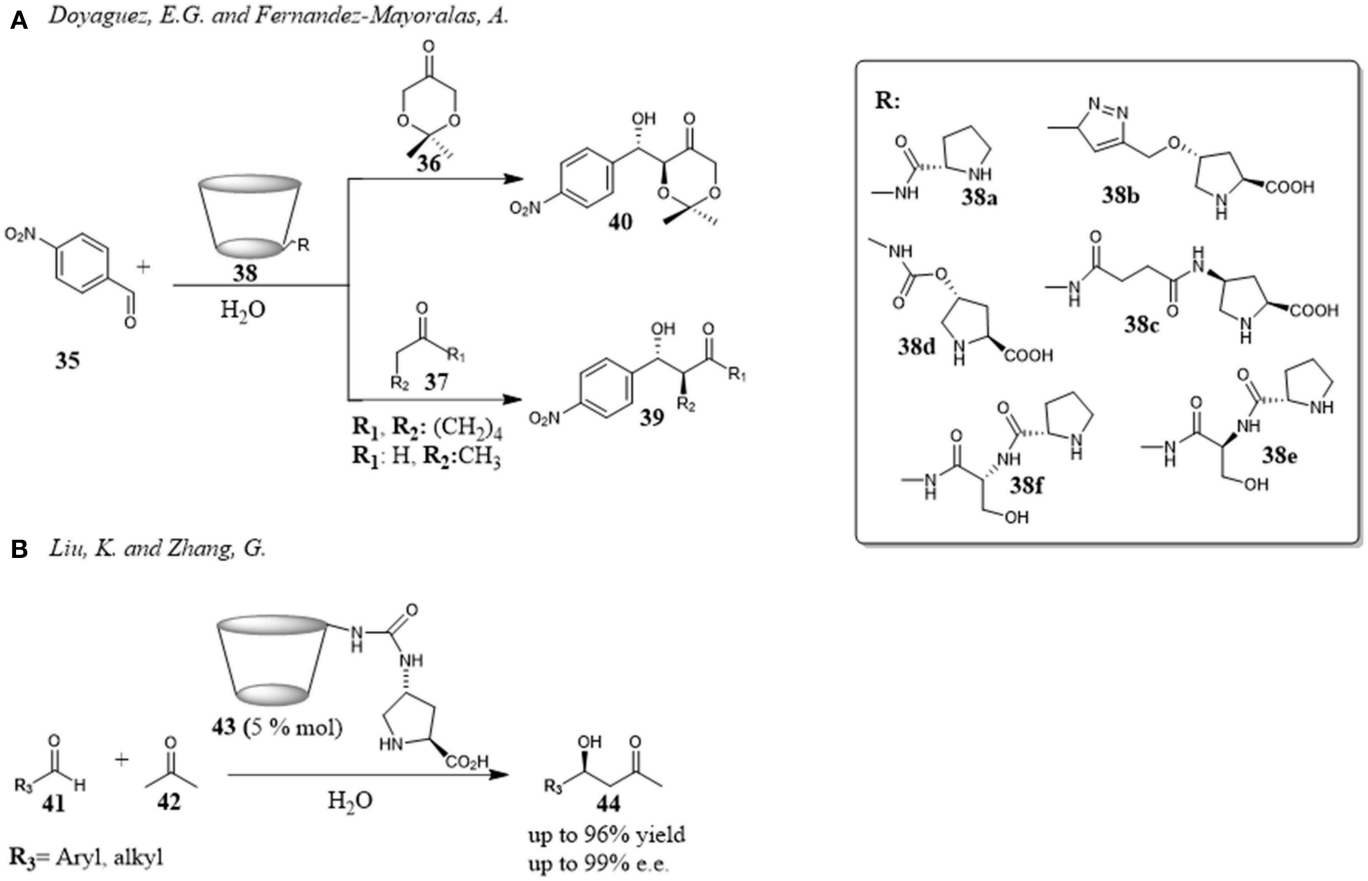
Proline-cyclodextrin coniugates catalyze the aldol reaction: **(A)** Aldol reaction between p-nitrobenzaldehyde and different ketones in the presence of CD-derivatives 38; **(B)** Aldol reaction between different aldehydes and acetone in the presence of CD-derivative 43.

In 2012, Doyagüez and Fernández-Mayoralas reported the use of cyclodextrins functionalized with proline as efficient supramolecular organocatalysts for aldol reactions in water (Figure 8A). Several coniugates of L-proline and β-CD **38** were prepared changing the nature of the linker between proline and β-CD and tested as catalysts for the aldol reaction between p-nitrobenzaldehyde **35** and different ketones **36** and **37**. A dependency on the nature of the linker was highlighted and the best results were obtained with more flexible succinamyl moiety. Studies were performed to understand the role played by β-CD. The reaction carried out with the proline arm alone, depleted of CD scaffold, did not take place even after prolonged reaction times, and the poor conversion observed by adding β-CD to the reaction suggested a role of β-CD in dissolving the reactants. The reaction efficiency decreased by adding sodium 2-naphthalenesulfonate, a known high affinity guest for β-CD (Inoue et al., 1993), pointing out the role of hydrophobic cavity by forming inclusion complexes with the aldehyde. Unfortunately, although the reaction efficiency ranged from moderate to high yields, the enantioselectivity was poor (Doyagüez and Fernández-Mayoralas, 2012).

Higher enantioselectivities were obtained by Liu and Zhang for the aldol reaction between different aldehydes **41** and acetone **42** in the presence of a β-cyclodextrin **43** with a proline linked via a urea functional unit (Figure 8B). Aromatic and aliphatic aldehydes reacted with acetone in moderate to good yields and with good to high enantioselectivities. The reaction efficiency showed a substrate dependence that the authors attributed to the different binding abilities with the interior cavity of β-CD. In fact, the drop in the reaction enantioselectivity obtained in the order para-, meta- and orto-substituted benzaldehydes was consistent with the decrease of binding constants between the substituted aromatic aldehydes and β-CD. Moreover, an interesting advantage of the procedure was the recycle of the catalyst. After the product isolation with an organic solvent, the catalyst being soluble in water remained in the aqueous phase which could be used as reaction medium for another reaction. This process could be done several times without loss of the reactivity and enantioselectivity. (Liu and Zhang, 2015).

**Figure 9:**
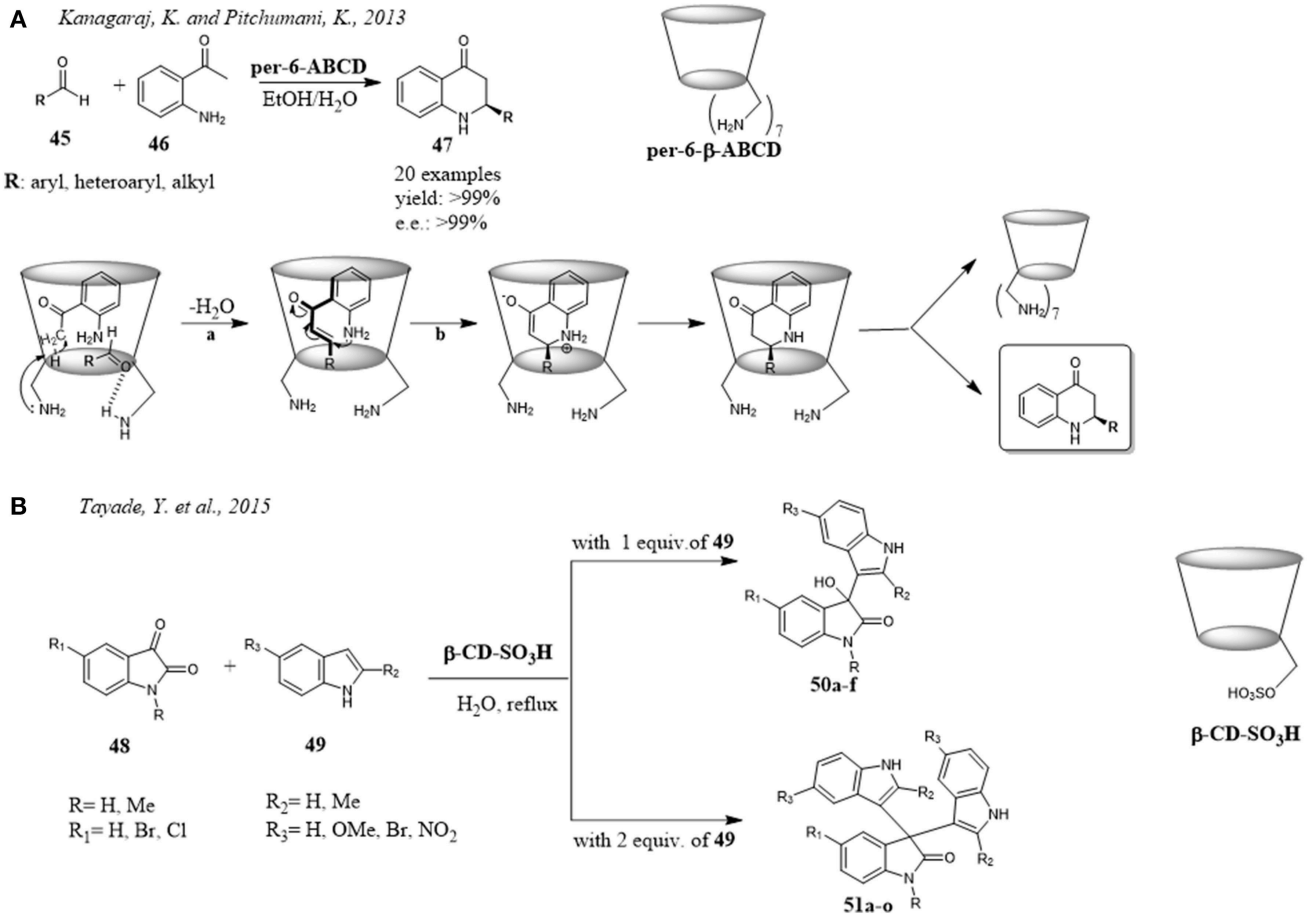
Synthesis of heterocycles promoted by CDs: **(A)** Peramino-CDs promote the synthesis of quinolone scaffolds; **(B)** Efficient synthesis of indole derivatives catalyzed by a sulfonated-β-cyclodextrin.

In 2013, β-cylodextrins peramino (per-6-ABCDs) functionalized on primary face were reported for the first time in an efficient one-pot procedure for the synthesis of enantiomerically enriched quinolone derivatives **47**, privileged building blocks useful to access a wide array of biologically active compounds (Kanagaraj and Pitchumani, 2013). Per-6-ABCDs behaved simultaneously as a supramolecular host and a chiral multifunctional base catalyst giving the reaction adducts with high yields and enantiomeric excesses. The per-substitution of the CD with amino groups was demonstrated to be necessary for the reaction outcome. When the reaction was performed with native β-CD or β-CD and external base or per-amino-β-CD hydrochloride, the reaction efficiency fell down significantly in terms of conversion and enantioselectivity. The substrate scope of the reaction was explored using various aryl and heteroaryl substituted aldehydes **45**. The reaction worked well showing independent on the electronic nature of aryl substituents, but their position on the ring and size exerted significant influence on the enantioselectivity suggesting the importance of a tight fitting of guests within the chiral cavity. The mechanism proposed involved in the first step the inclusion of *o*-aminoacetophenone **46** and aldehyde **45** within CD cavity. The primary amino groups of CD acted both as a base abstracting a proton by the acetyl group of *o*-aminoacetophenone **45** and hydrogen-bond donor toward the carbonyl group of aldehyde **46** (Figure 9A). The condensation between the activated substrates yielded the corresponding chalcone which subsequently underwent isomerization via aza-Michael reaction and then tautomerization to give the target compounds. The inclusion of *o*-aminoacetophenone **45** inside the CD cavity and its activation at *Si* face by amino groups of CD drove the stereochemical outcome of the aza-Michael addition. Excellent enantiomeric excesses were obtained with a host to guest ratio of 1:1. Molecular modeling studies, NMR and ESI-MS data provided further support to the proposed mechanism.

Another efficient application of modified CDs in the synthesis of heterocyclic scaffolds was reported by Tayade et al. A sulfonated-β-cyclodextrin was able to promote the reaction between different indoles **49** and isatins **48** to give the corresponding products **50** or **51** in good to excellent yields in very short reaction times (Figure 9B). The results obtained showed that the electronic effects of the substituents influenced only slightly the reaction outcome. The accelerated rate and high efficiency were ascribed to greater solubility of sulfonated-β-CD than native β-CD in water and more effective formation of inclusion complexes with isatins. Reaction optimization studies highlighted that 10 mol% of sulfonated-β-cyclodextrin and reflux temperature were the best conditions for this reaction resulting in excellent yields and extremely short reaction times (from 5 h at room temperature to 5 min at reflux). Additional advantage of the reaction was that the catalyst could be recovered and reused for three catalytic cycles without significant loss in catalytic activity (Tayade et al., 2015).

**Figure 10:**
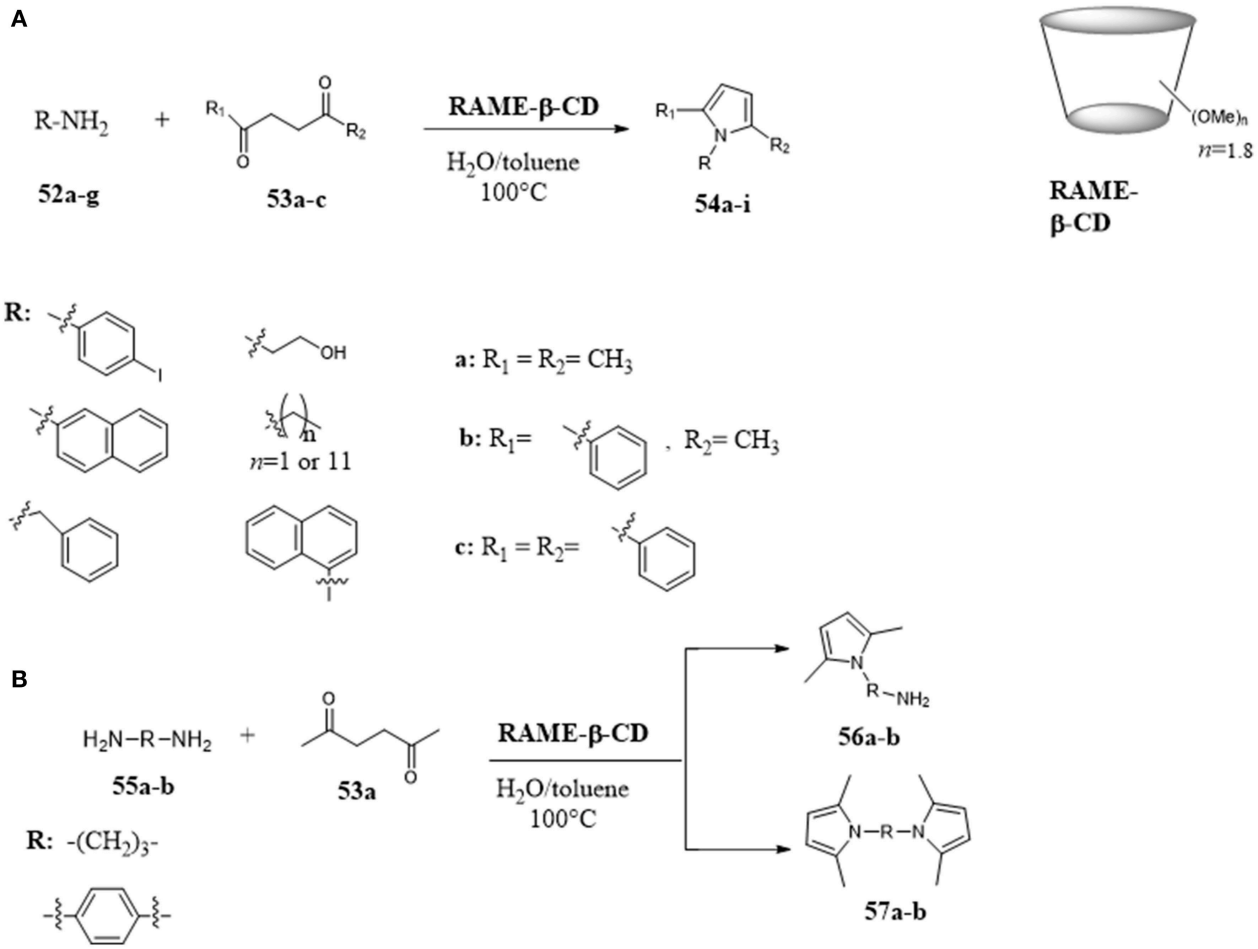
Paal-Knorr pyrrole synthesis assisted by partially methyled β-cyclodextrins: **(A)** Paal-Knorr condensation of 1,4-diketones and amines; **(B)** Paal-Knorr condensation of 1,4-diketones and diamines.

Amazing results were obtained using cyclodextrins in the Paal-Knorr condensation between 1,4-diketones and primary amines for the synthesis of pyrroles. (Menuel et al., 2014; Akelis et al., 2016) Partially methylated β-CDs (RAME-β-CD) have been used as mass transfer agents promoting the Paal-Knorr condensation of 1,4-diketones **53** and amines **52** for the synthesis of *N*-substituted pyrroles **54** under mild reaction conditions (Figure 10A). The activity of RAME-β-CDs resulted better than their native analogs and the results were explained on the basis of their higher water solubility and surface activity. The reaction was explored with different ketones and aromatic and aliphatic amines. In all cases, the corresponding products were obtained in good yields. The proposed mechanism involved the inclusion of amine inside the CD cavity, and an interaction between the amine and an hydroxyl group of the CD which promoted pyrrole formation. Particularly, when the reaction was performed with aliphatic and aromatic diamines **55**, the amount of amine used affected the selective formation of monopyrrole **56** or unsymmetrical bis-pyrrole derivatives **57** (Figure 10B).

### Calixarenes

Calixarenes represent a fascinating class of macrocyclic compounds particularly investigated in supramolecular chemistry (Figure 1B). They are constituted by *p-tert*-butylphenol units bridged with methylene units. Calix[*n*]arenes have the general structure reported in Figure 1 in which the number *n* of phenolic units ranges from 4 to 20. However, the most studied members are those constituted by 4–8 aromatic units. The name “calixarene” was introduced by C. D. Gutsche who was inspired by the similarity of the cone-conformation of calix[4]arene **1a** with a greek vase (*calix crater*) (Gutache et al., 1975; Gutsche and Muthukrishnan, 1978). Subsequently, this name was also extended to the other calixarene homologs. Calixarenes show a central hydrophobic cavity of controllable size, in addition to a wide upper rim and a narrow lower rim. The synthetic and structural versatility of these macrocycles make them suitable for different applications, (Böhmer, 1995; Gaeta et al., 2002; De Rosa et al., 2015; Neri et al., 2016; Talotta et al., 2017) including molecular recognition and sensing (Guo et al., 2011, 2012, 2013; Gaeta et al., 2012; Guo and Liu, 2014; Sun et al., 2016; Talotta et al., 2016; Yeon et al., 2016; Zheng et al., 2018), self-assembly processes (Rebek, 2000; Rudkevic, 2001; Díaz- Moscoso et al., 2016), synthesis of interpenetrated architectures (Gaeta et al., 2013, 2016; Arduini et al., 2016; De Rosa et al., 2017c; La Manna et al., 2017; Talotta et al., 2018), and catalysis (Cacciapaglia and Mandolini, 2000; Li et al., 2011; Deraedt and Astruc, 2016; Stoikov et al., 2016; Yilmaz and Sayin, 2016).

**Figure 11:**
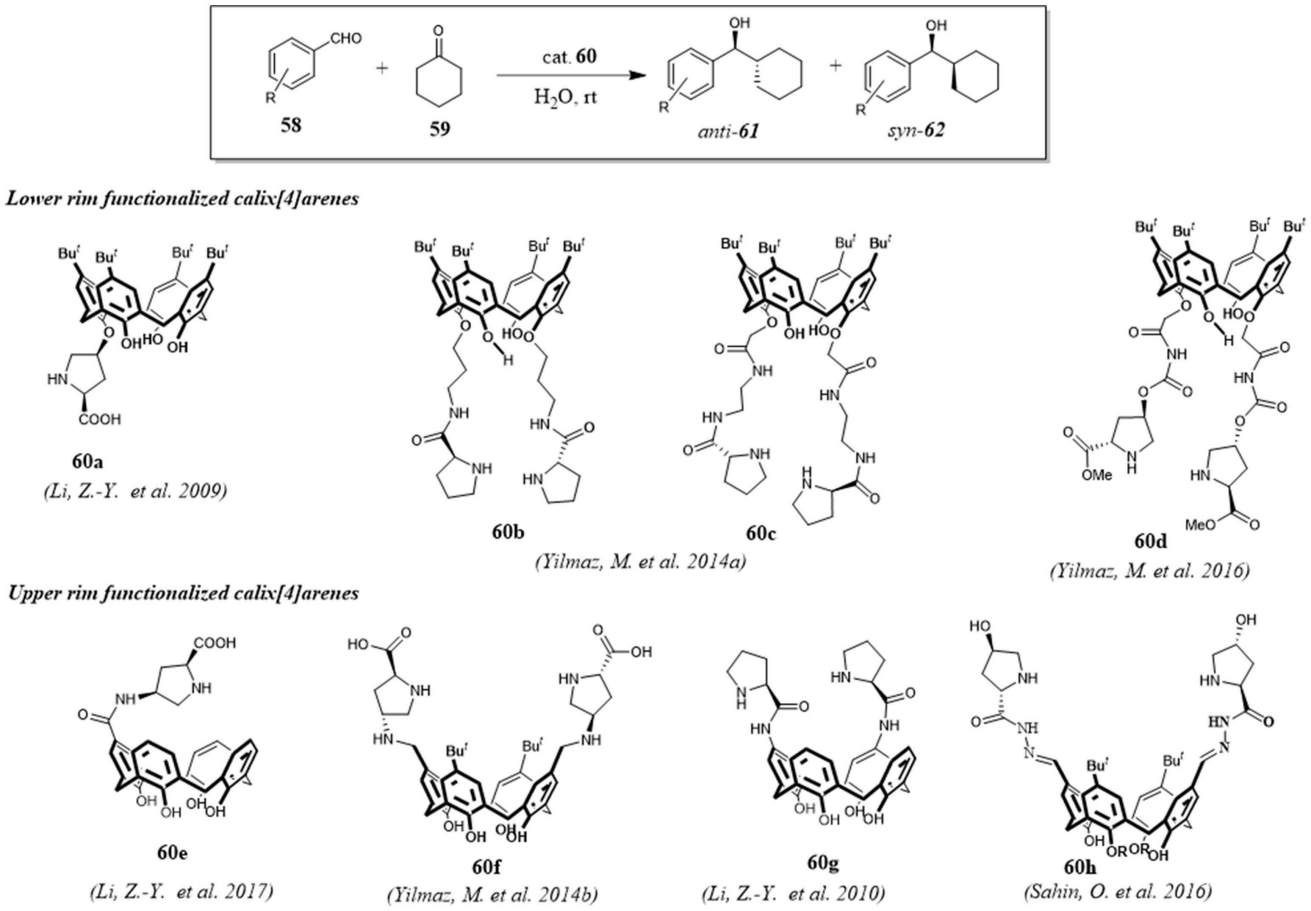
Direct asymmetric aldol reaction catalyzed by calix[4]arene functionalized with a chiral proline.

Calix[4]arenes bearing a chiral proline moiety at upper or lower rim have been successfully used by some research groups as organocatalysts for the direct aldol reaction between cyclohexanone **59** and various aromatic aldehydes **58** in water. (Figure 11) The catalytic activity of these calixarene derivatives highlighted the key role of their hydrophobic cavities for the reaction outcome, considering the poor activity of proline alone as promoter of the direct aldol reaction in water. For all catalysts the observed results suggested a mechanism based on general enamine catalysis where the reaction was accelerated by the formation of a hydrophilic and hydrophobic region through hydrogen bonding interactions between the free-OH groups of interfacial water molecules and the H-bond acceptor groups such as OH, NH, and CO groups of the calixarene scaffolds. Under optimal conditions, from good to high yields (>95%), high enantioselectivities (>90%) and from moderate to high diastereoselectivities (up to 65/35) were observed for all organocatalysts **60a-h**. The recycle of the catalysts and their reuse without notable loss of activity and selectivity were characteristics common to all catalysts. Interestingly, catalyst **60e** showed selectivity for cyclic ketones with different size thus providing further proof of the usefulness of its cavity as a selective nanoreactor for reactants (Li et al., 2009, 2010, 2017; Eymur et al., 2014; Uyanik et al., 2014; Aktas et al., 2016; Sahin et al., 2016).

**Figure 12:**
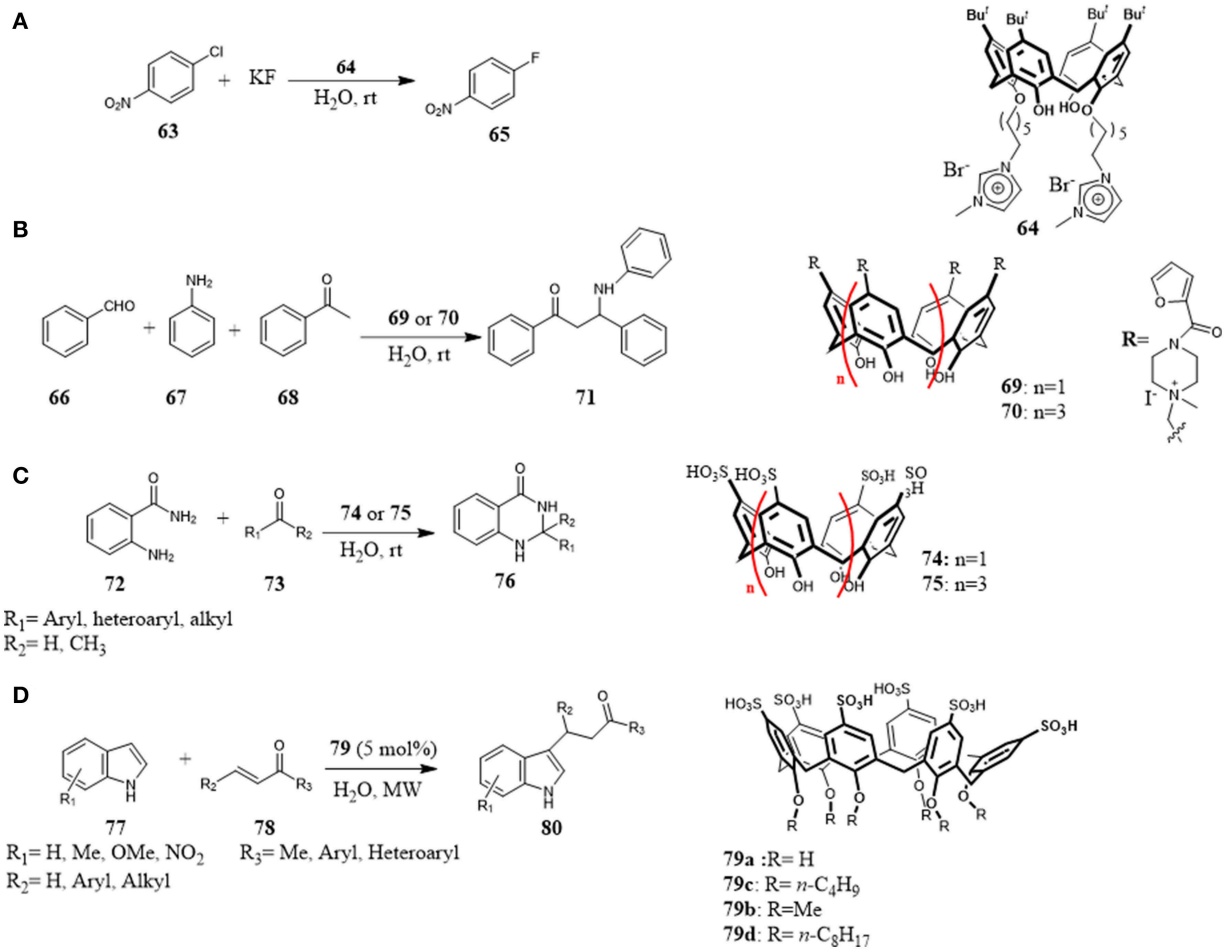
Water-soluble calixarenes as supramolecular organocatalysts in: **(A)** nucleophilic substitution reaction; **(B)** Mannich reaction; **(C)** cyclocondensation reaction; **(D)** Michael addition reaction.

A calix[4]arene bearing at lower rim imidazole moieties (**64**) was reported as an efficient phase-transfer catalyst for aromatic nucleophilic substitution reaction and benzyl nucleophilic substitution (Figure 12A). The high catalytic abilities compared to classical ionic liquids suggested that not only the good solubility in water due to the presence of ions groups but also the combination with complexation abilities of its cavity played an important role for the catalysis: the aromatic substrate solubility in water was improved by inclusion in the calixarene cavity and by its structural conformation. The catalytic activity of the calixarene derivative was higher with the calixarene in more stable cone conformation (Yang et al., 2012).

In 2014, Sayin and Yilmaz investigated the feasibility to use calix[n]-arenes functionalized at the upper rim with quaternary ammonium salts **69** and **70** as catalyst for one-pot Mannich reaction between benzaldehyde **66**, acetophenone **68** and aniline **67** in water. They found that the calixarene catalysts gave β-aminocarbonyl compounds **71** in high yields and very short reaction times, even with small amount of catalyst. (Figure 12B) Studies on the effect of cavity were carried out highlighting its involvement in the catalysis. In the presence of tetrabutylammonium bromide (TBAB) as competitive guest for the cavity, no conversion to reaction products was observed, even after prolonged reaction times, thus suggesting that the reaction occurred inside the cavity. Furthermore, comparing the results obtained with calix[4]- and calix[6]arene, it was highlighted that the calix[6]arene with a larger cavity size worked better than the other one in terms of yields and reaction times. Therefore, it could be concluded that the reaction was triggered by the host-guest complexation of benzaldehyde with calixarene, and the furoyl-piperazinium groups at the upper rim interacted with aniline and acetophenone by weak non-covalent interactions to bring closer to the benzaldehyde (Sayin and Yilmaz, 2014).

In 2015, Rahman et al. used p-sulfonic acid calixarenes **74** and **75** as organocatalysts for the synthesis of 2,3-dihydroquinazolyn-4(1H)-ones **76** in water (Figure 12C). The reaction of cyclocondensation between anthranilamide **72** and different carbonyl compounds **73** afforded the target compounds in high yields under mild reaction conditions. The reaction performed with p-hydroxy benzenesulfonic acid (p-HAS) as catalyst, resulted less efficient than the reaction with calixarene derivatives **74** underlining the role of calixarene framework combined with its ability to activate aldehyde via H-bonding interaction with the sulphonic groups on the reaction. The general scope, the scale-up of the reaction to gram-scale, easy work-up, low amount of catalyst, mild reaction condition, and short reaction time are considerable advantages of this protocol (Rahaman et al., 2015).

Another example of calixarene sulfonic acid derivatives as efficient organocatalysts has been described by Xie et al. Alkylation of indoles **77** with α,β-unsaturated ketones **78** by means of Michael addition was promoted by water-soluble calix[6]arene sulfonic acids under microwave irradiation in water (Figure 12D). Calix[6]arene sulfonic acids **79** acted as inverse phase transfer catalysts. Particularly, calixarene **79d** bearing pendant aliphatic chains and thus offering the possibility to form micelles in water, proved to be the optimal catalyst. The scope of the reaction was broad with respect to different indoles **77** and α,β-unsaturated ketones **78**, low amount of catalyst (5 mol%) are able to promote the reaction affording the Michael adducts in good to high yields (Xie et al., 2013).

**Figure 13:**
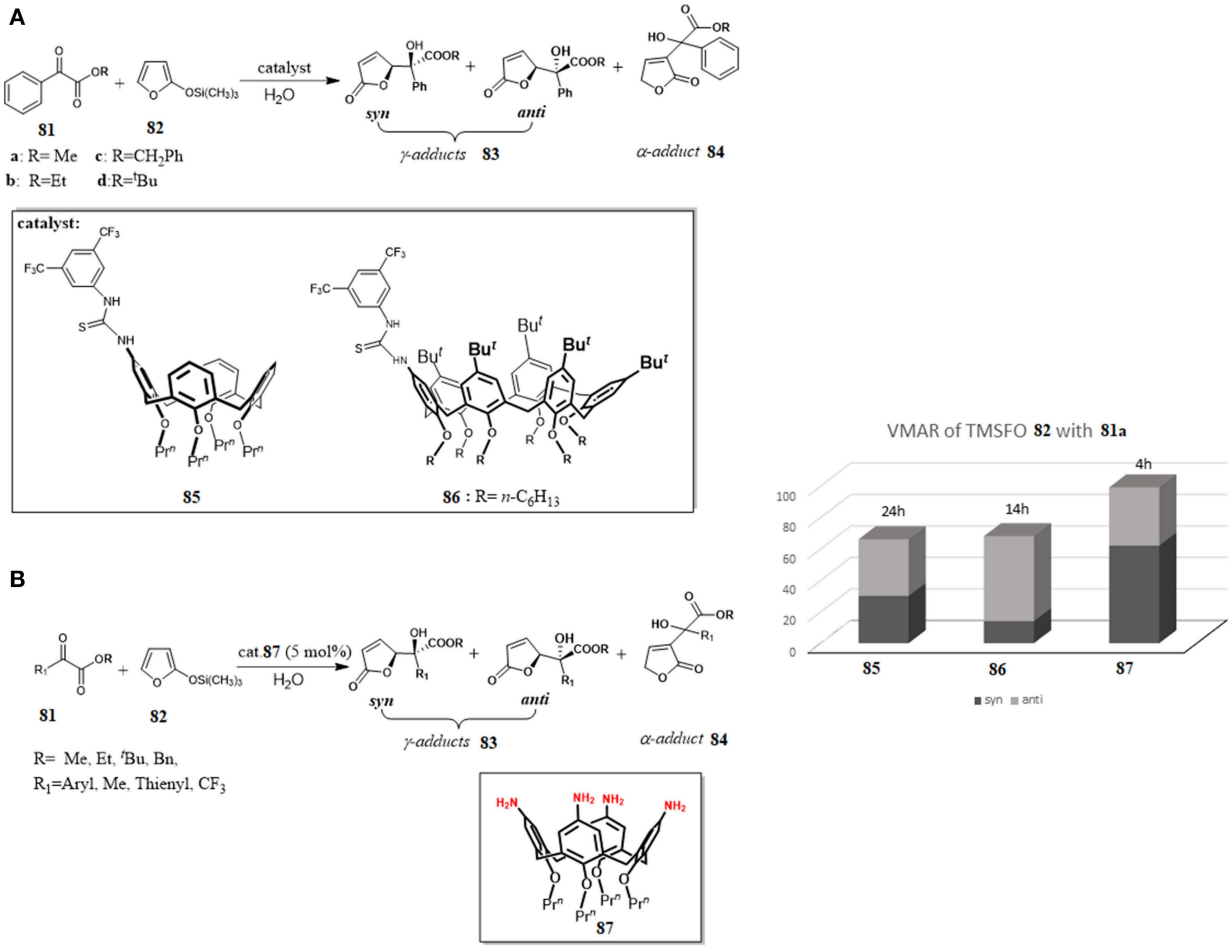
Calixarenes as efficient organocatalyst for a vinylogous Mukaiyama aldol reaction (VMAR) under “on-water” conditions: **(A)** VMAR promoted by thioureido-calixarene derivatives 85 and 86; **(B)** VMAR catalyzed by tetraminocalix[4]arene 87.

Recently, calix[n]arene scaffolds adorned with a supramolecularly interacting group such as thiourea moiety have been used as organocatalysts for Vinylogous Mukaiyama Aldol reaction (VMAR) between 2-(trimethylsilyloxy)furan **82** and α-ketoesters **81** under “on-water” conditions (Figure 13A). The reaction is a useful protocol to obtain functionalized γ-butenolides, which are valuable building blocks for many biologically active compounds and, recently interesting for the engineering of new crystalline assemblies (De Rosa et al., 2009, 2017a). It was shown that the catalytic ability of the calixarenes **85** and **86** was closely related to their remarkable hydrophobicity and their recognition abilities toward the reactants through hydrogen—bonding interactions with a thioureido group at the upper rim. The catalysts were more active using water as reaction medium than other organic solvents, the reaction was completely regioselective affording only the γ-adducts **83** and, interestingly, a switch of stereoselectivity was observed going from organic solvent to water with a preference for *anti* diastereoisomer **83**. It is noteworthy that, by increasing the dimension of the calixarene-catalyst (**85** vs. **86**), and consequently its hydrophobicity, a significant increase of the reaction rate occurred. Binding studies between catalyst and α-ketoesters by ^1^H-NMR spectroscopy and molecular mechanics calculations clearly indicated a supramolecular control of this catalysis associated with recognition abilities of calixarenes (De Rosa et al., 2016).

In a later paper, the same authors proposed that the hydrophobic amplification observed under “on- water” conditions could enable even weaker H-bond donor moieties to catalyze the VMAR under “on-water” conditions (De Rosa et al., 2017b). In fact, simple tetraminocalix[4]arene **87** with weak H-bond donor NH_2_ groups was an efficient organocatalyst for the reaction of TMSFO **82** and α-ketoester **81** under “on-water” conditions and its catalytic activity was superior to that in organic solvents. Furthermore, its activity was better than previous catalysts **85** and **86** in terms both conversion, reaction times and selectivity and there was a switch of the stereoselectivity in favor of *syn*-adduct (Figure 13). The reaction was general with a variety of α-ketoesters with good to high yields and showed in all cases a preference for *syn* diastereomer *syn* **83**. The reaction outcome was rationalized through a multipoint recognition model where the amino groups of the calixarene were engaged in H-bond interactions with both reactants favoring the stabilization of a ternary complex **81**·**82**·**87** that drove the attack at the activated carbonyl group of α-ketoesters on a favored side. The proposed model was in agreement with the differences in the conversion and stereoselectivity detected with different substrates.

## Conclusion

In the last years, significant advances have been made in the field of supramolecular organocatalysis using water as a reaction medium and thus demonstrating its synthetic potential. Different types of reactions promoted by supramolecular organocatalysts in water have been reported, thus providing not only environmentally friendly and efficient procedures, but in some cases new reactivity and reaction pathways different from those in organic solvents. Two principal topics drive this type of catalysis: (1) the hydrophobic effect and (2) the molecular recognition. Here, we have focused our attention on selected recent developments in this field using cyclodextrins and calixarenes as supramolecular scaffolds. These macrocycles are interesting because they provide a hydrophobic cavity, with tunable size and shape, capable of selectively accommodating the substrates isolating them from the reaction environment. In addition, the easy functionalization of their scaffold allows to introduce additional catalytic groups or binding sites in specific positions. The reported examples highlight how the combined action of these two characteristics have a positive effect on the reaction outcome.

In analogy to natural enzymes which have evolved over time improving efficiency and selectivity, we think that supramolecular organocatalysis in water can be expected to grow in the coming years demonstrating a further broadening of its applications.

## Author contributions

MD: Wrote manuscript and chose references; PL: Gave assistance for literature search; CT: Provided assistance for some revisions; AS: Contributed to the discussions and gave assistance for literature search; CG: Designed the main content; PN: Provided professional advice. All authors read and approved the final manuscript version to be submitted.

### Conflict of interest statement

The authors declare that the research was conducted in the absence of any commercial or financial relationships that could be construed as a potential conflict of interest.
